# Modeling Parkinson’s Disease Through Combined DJ-1 Deficiency and Noradrenergic Degeneration

**DOI:** 10.3390/cimb48101047

**Published:** 2026-10-10

**Authors:** Filipa Ferreira-Antunes, Ana Marote, Jonas Campos, Inês Feio Borges, Daniela Monteiro-Fernandes, Sandra Barata-Antunes, Bárbara Mendes-Pinheiro, António J. Salgado, Belém Sampaio-Marques

**Affiliations:** 1 Life and Health Sciences Research Institute (ICVS), School of Medicine, University of Minho, 4710-057 Braga, Portugal; id11400@alunos.uminho.pt (F.F.-A.); anamarote@med.uminho.pt (A.M.); jonascampos@med.uminho.pt (J.C.); a99895@alunos.uminho.pt (I.F.B.); danielamonteiro@med.uminho.pt (D.M.-F.); sandra.barata17@gmail.com (S.B.-A.); barbarafmpinheiro@gmail.com (B.M.-P.); 2 Life and Health Sciences Research Institute (ICVS)/Biomaterials, Biodegradables and Biomimetics Research Group (3B’s)—PT Government Associate Laboratory, 4710-057/4805-017 Braga/Guimarães, Portugal

**Keywords:** Parkinson’s disease, locus coeruleus, DJ-1, noradrenergic degeneration

## Abstract

Parkinson’s disease (PD) is characterized by progressive degeneration of the nigrostriatal dopaminergic system while increasing evidence indicates that early pathology also involves the locus coeruleus (LC) and the noradrenergic system. However, whether LC degeneration increases dopaminergic vulnerability in genetically susceptible background remains poorly understood. Here, we investigated the impact of selective noradrenergic depletion on behavioral and neuropathological phenotypes in DJ-1 knockout (KO) mice, a model of familial PD. DJ-1 KO and wild-type (WT) mice received the selective LC neurotoxin DSP-4 (50 mg/kg) to induce noradrenergic degeneration, followed by a battery of motor and non-motor behavioral assessments and histological analysis of the LC and nigrostriatal system. DSP-4 induced approximately 80–85% LC neuronal loss, confirming effective noradrenergic lesioning. While DSP-4-treated WT mice displayed relatively mild behavioral alterations, its combination with DJ-1 deficiency produced marked impairments, including increased anxious- and depressive-like behaviors, enhanced contextual fear responses, and deficits in motor coordination and balance. At the nigrostriatal level, DJ-1 KO mice already exhibited reduced TH-positive neurons in the substantia nigra pars compacta (SNc), whereas combined DJ-1 deficiency and DSP-4 treatment produced a further significant reduction in striatal TH density, without significant additional loss of SNc TH-positive neurons. These findings indicate that LC noradrenergic degeneration selectively exacerbates dopaminergic terminal pathology in the DJ-1-deficient background, suggesting increased vulnerability of the nigrostriatal system to noradrenergic loss. Overall, our data identify LC degeneration as a modifier of both motor and non-motor phenotypes associated with DJ-1 deficiency and provide experimental support for a functional interaction between the noradrenergic and nigrostriatal systems in PD.

## 1. Introduction

Parkinson’s Disease (PD) is the most common movement disorder and the second-most common neurodegenerative disorder, affecting more than 10 million people worldwide [1]. PD is pathologically characterized by degeneration of dopaminergic neurons in the substantia nigra pars compacta (SNpc) and dopamine depletion in the striatum (STR), leading to changes in the functioning of downstream basal ganglia circuitry and consequent motor impairments [1]. Recently, an increasing body of knowledge has shown that before the appearance of motor symptomatology, patients experience non-motor symptoms and subtle motor alterations [2]. This prodromal stage of PD has been correlated with degeneration in other neurotransmitter systems in addition to the dopaminergic nigrostriatal pathway such as the noradrenergic system [1,2]. A structure that has been proposed to be early affected in PD is the locus coeruleus (LC) [3,4]. This brainstem nucleus is the major source of noradrenaline, involved in various functions, including regulation of arousal, attention, and stress responses [3,4,5,6]. LC degeneration has been correlated with the development and aggravation of both motor and non-motor PD symptoms [3,7,8,9]. Nevertheless, additionally to understand the degenerative processes and regions affected during PD, it is important to acquire insight into how genetic mutations may influence vulnerability to degeneration.

Genetic factors have been increasingly recognized as part of PD etiology [10]. Investigating the genetic basis of PD may provide insights into disease mechanisms, risk assessment, early diagnosis, and the development of targeted therapies. Autosomal recessive mutations in the PARK7 gene, which encodes for the DJ-1 protein, accounts for about 1–2% of early-onset PD cases. DJ-1 is a highly conserved protein (189 amino acids) that regulates numerous processes important in neuroprotection, such as anti-oxidative, anti-apoptotic and anti-inflammatory pathways [10,11,12]. Patients with DJ-1 mutations exhibit an early onset of rigidity, tremors, dyskinesia and generally have a good response to levodopa (L-DOPA) treatment [11,12,13,14]. Moreover, DJ-1-linked PD cases often present non-motor symptomatology, such as anxiety and cognitive deficits, and have shown early-onset degeneration in the LC [11,12,14]. Notably, LC degeneration has also been reported in individuals with DJ-1-associated PD [11,12,14], suggesting that noradrenergic pathology may be particularly relevant in the context of DJ-1 deficiency. Consistent with this, DJ-1 knockout (KO) mice exhibit increased susceptibility to cellular stress and alterations in dopaminergic function, providing an experimental model in which genetic vulnerability can be investigated [15].

Still, a central challenge in PD research is to understand how distinct pathological factors interact to determine the selective vulnerability of neuronal populations. Although DJ-1 deficiency and LC degeneration have each been investigated as contributors to PD-related pathology, the consequences of combining a genetic susceptibility factor with selective noradrenergic degeneration remain poorly characterized. In particular, it is not clear whether LC degeneration can exacerbate nigrostriatal dysfunction in a DJ-1-deficient background, nor whether such an interaction preferentially affects dopaminergic neuronal cell bodies or their striatal terminals. Addressing this question may provide insight into how multisystem degeneration contributes to the emergence of both motor and non-motor manifestations of PD.

To investigate this interaction, we combined a DJ-1 knockout mouse model with selective LC noradrenergic degeneration induced by the neurotoxin DSP-4. This approach was designed to model the interaction between genetic vulnerability and an additional pathological insult affecting the noradrenergic system, involved in the prodromal and clinical manifestations of PD. We hypothesized that selective LC degeneration would increase the vulnerability of the nigrostriatal system in DJ-1 KO mice, resulting in exacerbated behavioral and neuropathological alterations compared with either manipulation alone. We, therefore, assessed motor and non-motor behaviors and characterized LC and nigrostriatal pathology following DSP-4 administration in DJ-1 KO and wild-type mice. By examining these effects across behavioral and anatomical levels, this study aims to clarify the contribution of noradrenergic degeneration to dopaminergic vulnerability in a genetically susceptible model of PD.

## 2. Materials and Methods

### 2.1. Animals

In this study, we used a DJ-1 KO (DJ-1^−/−^) mouse model established by Goldberg et al. [15]. Heterozygous (DJ-1^+/−^) male and female mice were kindly provided by his lab (Center for Neurologic Diseases, Harvard Medical School, Boston). From this couple, we have established a colony inhouse by crossing heterozygous DJ-1 male and female mice and performing genotyping of the offspring. Three-month-old DJ-1 mice and WT littermates, male and female, were used within the scope of project 005453, according to the Portuguese national authority for animal research, Direção Geral de Alimentação e Veterinária (DGAV; 69914/25S; 21 July 2025) and Ethical Subcommittee in Life and Health Sciences (SECVS; ID: SECVS 142/2016, University of Minho). Animals were housed in groups, obeying animal limits per cage, in a temperature- and humidity-controlled room, on 12 h light/dark cycles, and access to food and water ad libitum. Humane endpoints were predefined to minimise animal suffering during the study. Animals were monitored throughout the experimental period, and euthanasia would have been performed if an animal reached either a 20% reduction from its normal body weight or a 15% reduction in body weight occurring within a 2-day period. No animals reached the predefined humane endpoints during the study, and, therefore, no animals required euthanasia for this reason.

### 2.2. DSP-4 Administration and Experimental Design

Animals were randomly allocated to the experimental groups to minimize potential selection bias and ensure comparable distribution of animals across control and treatment conditions. Randomization was performed prior to the start of the experimental procedures. All experimental procedures involving outcome assessment and data analysis were performed under blinded conditions. The researchers responsible for performing and analyzing the experiments were unaware of the experimental group allocation throughout the assessment and analysis procedures. Animals were also housed independently of their experimental group to minimize the potential for group-related influences on experimental procedures and outcome assessment. Group identities were only disclosed after completion of the respective analyses. N-(2-chloroethyl)-N-ethyl-2-bromobenzylamine (DSP-4) (C8417, Sigma-Aldrich, St. Louis, MO, USA) was systemically injected by intraperitoneal injection (i.p.) at the defined dosage. A set of animals received the same volume of saline (NaCl), which was the vehicle used to prepare the DSP-4 solution. Animals (ntotal = 43) were randomly assigned to 6 groups (*n* = 5–11/group): (1) WT vehicle; (2) WT + DSP-4 50 mg/kg; (3) DJ-1 vehicle; (4) DJ-1 + DSP-4 10 mg/kg; (5) DJ-1 + DSP-4 25 mg/kg and (6) DJ-1 + DSP-4 50 mg/kg. The required sample size was determined a priori based on previously published data reporting effect sizes and variability associated with DSP-4 usage. Sample size calculations were performed using G*Power version 3.1.9.7, with an effect size of 0.45 and statistical power set at 0.95. The calculation accounted for four repeated measurements per animal, allowing for robust detection of treatment effects while minimizing the number of animals used in accordance with the principles of the 3Rs.

### 2.3. Behavioral Tests

**Figure 1:**
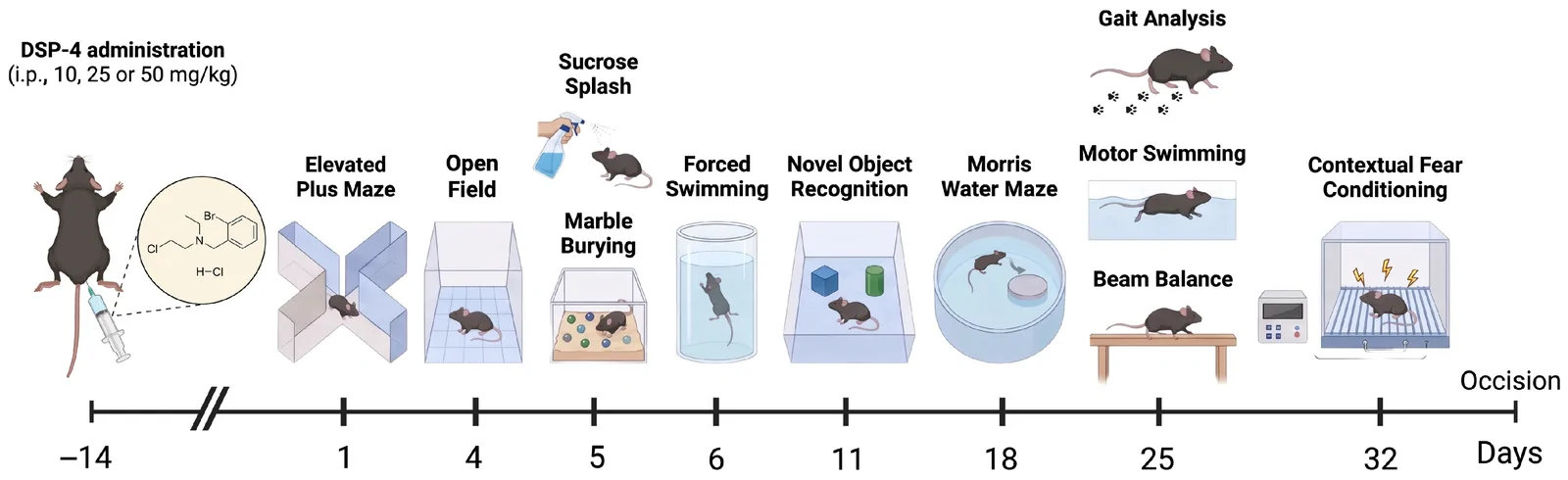
Experimental design. First, animals were separated into groups and injected with either saline or DSP-4. During the 2 weeks following injection, animals were weighed and welfare was assessed every 2 days. Then, behavioral assessment was started with tests evaluating anxiety- and depressive-like behaviors: Elevated Plus Maze (EPM), Open Field (OF), Marble Burying, Sucrose Splash Test (SST), and Forced Swimming Test (FST). In the following two weeks, we evaluated memory and learning paradigms: Novel Object Recognition (NOR) and Morris Water Maze (MWM). In the fourth week, we assessed motor coordination and balance: Beam Balance, Motor Swimming Test, and Gait Analysis. At week 5, we evaluated memory and learning using Contextual Fear Conditioning (CFC).

Behavioral assessments were conducted at the predefined time points of the experimental protocol to evaluate motor and non-motor alterations. The specific procedures used for each behavioral test are described in detail in the following subsections, according to the experimental timeline and study design presented in Figure 1.

#### 2.3.1. Elevated Plus Maze (EPM)

EPM was carried out under a bright white light in an apparatus consisting of a black propylene plus-shaped platform elevated 74.4 cm above the floor with two open arms (50.8 cm) and two closed arms (10.2 cm). Animals were placed individually in the center of the platform, and all sessions were video recorded over 5 min and manually analyzed using Kinoscope software (V.3.0.4, SourceForge, San Diego, CA, USA). The time animals spent in each arm and the number of entries in each arm were used as measures of anxiety-like behavior.

#### 2.3.2. Open Field (OF)

Animals were placed in the center of an illuminated square arena (43.2 cm × 43.2 cm) enclosed by a 30.5 cm high wall. Movements were tracked for 5 min, using a 16-beam infrared system (MedAssociates Inc., St Albans, VT, USA), and data were analyzed using Activity Monitor software (4v, Med Associates, Inc., St Albans, VT, USA). The distance traveled and time spent at the center versus the periphery were considered a measure of anxiety-like behavior.

#### 2.3.3. Marble-Burying

Each mouse was placed in a cage with six marbles for 30 min. The number of marbles buried was used as an index to evaluate anxiety-like behavior. A marble was considered buried if at least two-thirds of it was hidden in the bedding.

#### 2.3.4. Sucrose Splash Test (SST)

Animals were placed in a clean empty cage, and a 10% sucrose solution was sprayed on the dorsal coat of mice. The sucrose solution is usually chosen as a releaser and an enduring factor of grooming due to its viscosity. All sessions were video recorded over 5 min and analyzed manually using the Kinoscope software. The latency time to initiate a grooming, time spent grooming and number of groomings initiated were used as an index to measure depressive-like behavior.

#### 2.3.5. Forced Swimming Test (FST)

Each animal was individually placed in glass cylinders filled with water (30 cm of depth; 22 °C). All sessions were video recorded for 7 min total, and the first 2 min were considered an habituation period. Each session was analyzed manually using the Kinoscope software. The immobility time (IT), or total time spent immobile, was measured. Mice were considered immobile when remaining passively floating or performing the minimal movements needed to maintain their head above the surface.

#### 2.3.6. Morris Water Maze Test (MWM)

The test was performed in a circular swimming pool (150 cm diameter, 50 cm depth, 22 °C), divided into four quadrants, with a mobile target platform set about 1–1.5 cm under water surface. During four consecutive days, each animal had four trials, each one starting in a different quadrant. They were given 1 min maximum to find the platform. The time spent and the traveled distance to reach the platform was used as a measure of hippocampal-dependent learning. The average speed in each session was used to assess whether the results were correlated with motor impairments. All sessions were video recorded and analyzed using EthoVision XT 13 software (Noldus, Wageningen, The Netherlands).

#### 2.3.7. Balance Beam Test (Beam)

Mice were placed in an elevated, open platform, connected by a beam to a closed box and trained for 3 days on a square beam with 12 mm of diameter. On day 4, animals were tested on a large square beam (12 mm), on a round large beam (17 mm) and on a thin square beam (11 mm). The time animals took to cross each beam was registered. Whenever the animal stopped, time was discounted. If the animal fell or turned around it was considered a failed trial. Each animal had two opportunities to fail; otherwise, it was not included in the analysis.

#### 2.3.8. Motor Swimming Test

Animals were trained for two consecutive days (three trials per animal) to traverse a clear Perspex water tank (100 cm long, 22 °C) to a safe black platform at the end. Mice were tested for three consecutive days (two trials per animal), and the latency to cross the water was measured from the 60 cm distance mark.

#### 2.3.9. Gait Analysis

Mice’s hind and forepaws were coated with black or red non-toxic paints, respectively, before walking along an inclined corridor (100 cm × 4.2 cm × 10 cm), containing a clean paper sheet on the bottom. The stride width was obtained by manually measuring the distance between two pawprints. Three values were measured for six consecutive steps, and the mean of three values was used.

#### 2.3.10. Contextual Fear Conditioning (CFC)

The protocol herein employed used a light as a cue. A total of 20 s after the light turned on, animals received a 2 s shock (0.7 V), in 3 min intervals, during 9 min total. On the second day, animals were subjected to the same context, and when the light turned on, the responsiveness of mice, freezing, could be used as an index of contextual fear conditioning. Two hours later, on the second day, animals were placed in a different context and the responsiveness was analyzed. All sessions were video recorded and analyzed manually using Kinoscope software.

### 2.4. Tissue Processing

After behavioral assessment, mice were anesthetized with a mixture of ketamine (150 mg/kg) and medetomidine (0.3 mg/kg) and were transcardially perfused with cold 4% paraformaldehyde (PFA) in phosphate-buffered saline (PBS). Brains were collected and transferred to a tube containing 4% PFA in PBS. After 48 h of incubation at 4 °C, brains were transferred to a solution of 30% sucrose in PBS containing 0.01% of sodium azide and kept at 4 °C until being processed. Brains were sectioned coronally using a vibratome (VT1000S, Leica, Wetzlar, Germany) at thickness of 40 µm.

### 2.5. Immunohistochemistry

Six series of coronal sections of striatum, SNpc and LC were selected for free-floating immunohistochemistry. Sections were initially treated with PBS 3% H_2_O_2_ for 20 min to block endogenous peroxidase activity, permeabilized in 0.3% PBS-Triton (PBS-T) for 10 min and then blocked in PBS with 10% fetal calf serum (FCS) for 1 h to prevent non-specific binding. Then, sections were incubated in PBS with 2% FCS anti-TH antibody (rabbit polyclonal IgG, 1:1000; Merck Millipore, Darmstadt, Germany) overnight at 4 °C. On the next day, brain sections were incubated for 30 min at RT with a biotinylated secondary antibody (goat anti-polyvalent, TP-125-BN, ThermoFisher Scientific, Waltham, MA, USA) and then during 30 min with the streptavidin–peroxidase solution (TP-125-HR, ThermoFisher Scientific). To detect the streptavidin–biotin immunoenzymatic complex, a substrate was given to this reaction, using 12.5 mg of 3,3’- diaminobenzidine tetrahydrochloride (DAB; D5905, Sigma-Aldrich) dissolved in 25 mL of Tris-HCl 0.05 M (pH = 7.6) with 62.5 µL of H_2_O_2_. Sections were then mounted on superfrost slides and air-dried for 24 h at RT before analysis.

### 2.6. TH+ Fibers Density Quantification

TH+ fibers in the striatum were imaged using brightfield illumination (SZX16, Olympus, Tokyo, Japan), and the optical density was measured using the ImageJ software (National Institute of Health, v1.48, Bethesda, MD, USA). Each image was first converted to 8-bit format. Then, using the regions of interest (ROI) manager, the dorsal striatal area was selected in both hemispheres, as well as three different background regions next to the striatal area. White and black values were inverted (black = 0, white = 255) and the selected settings for the analysis were area and mean grey value (average gray value within the selection). After that, the measurement was performed, and the values for striatal area and backgrounds were saved.

### 2.7. TH+ Cell Quantification

TH-positive cells in the SNpc and LC were visualized and counted using a bright-field microscope (BX51; Olympus, Center Valley, PA, USA) equipped with a digital camera (PixeLINK PL-A622, CANIMPEX Enterprises, Halifax, NS, Canada). Using the Visiomorph software (V2.12.3.0; Visiopharm, Hørsholm, Denmark) the boundaries of the SNpc and LC area were drawn and total TH+ cells were counted in both hemispheres.

### 2.8. Statistical Analysis

All data sets were tested for normality distribution with the Shapiro–Wilk test prior to any statistical analysis. Data are expressed as the mean ± standard error of mean (SEM) unless otherwise stated in the figure legends. Equality of variances and sphericity were measured using the Levene’s and Mauchly’s tests, respectively. A one-way ANOVA was used to assess the differences among three or more groups. For all analysis, upon confirmation of significant main effects, differences among group means were analyzed using the Tukey post hoc test. A value of *p* < 0.05 was considered statistically significant. Statistical analyses were performed using GraphPad Prism (version 10.2.1, GraphPad Software, Boston, MA, USA).

## 3. Results

### 3.1. Dose–Response Characterization of DSP-4 Reveals Non-Motor Phenotype Without Welfare Compromise in DJ-1 KO Mice

To select the most suitable dosage to induce a lesion in the LC, without compromising the animals’ welfare, we conducted a preliminary study to test three different dosages previously described in the literature for WT animals, 10 mg/kg, 25 mg/kg, and 50 mg/kg in the DJ-1 KO animals herein used. The body weight of each animal was assessed before DSP-4 administration and 2-, 4-, 8- and 20-days post-injection and compared with DJ-1 KO mice injected with saline (vehicle/control). Neither DSP-4 dosage induced a significant or concerning reduction in animal weights throughout the course of the experiment (Figure S1).

Noradrenergic degeneration in the LC is correlated with the establishment of non-motor symptoms (e.g., anxiety, depression and cognitive impairments) which are common features of prodromal PD stages [5,16,17,18]. Therefore, we started by characterizing non-motor alterations evoked by different dosages of DSP-4-induced noradrenergic degeneration.

**Figure 2:**
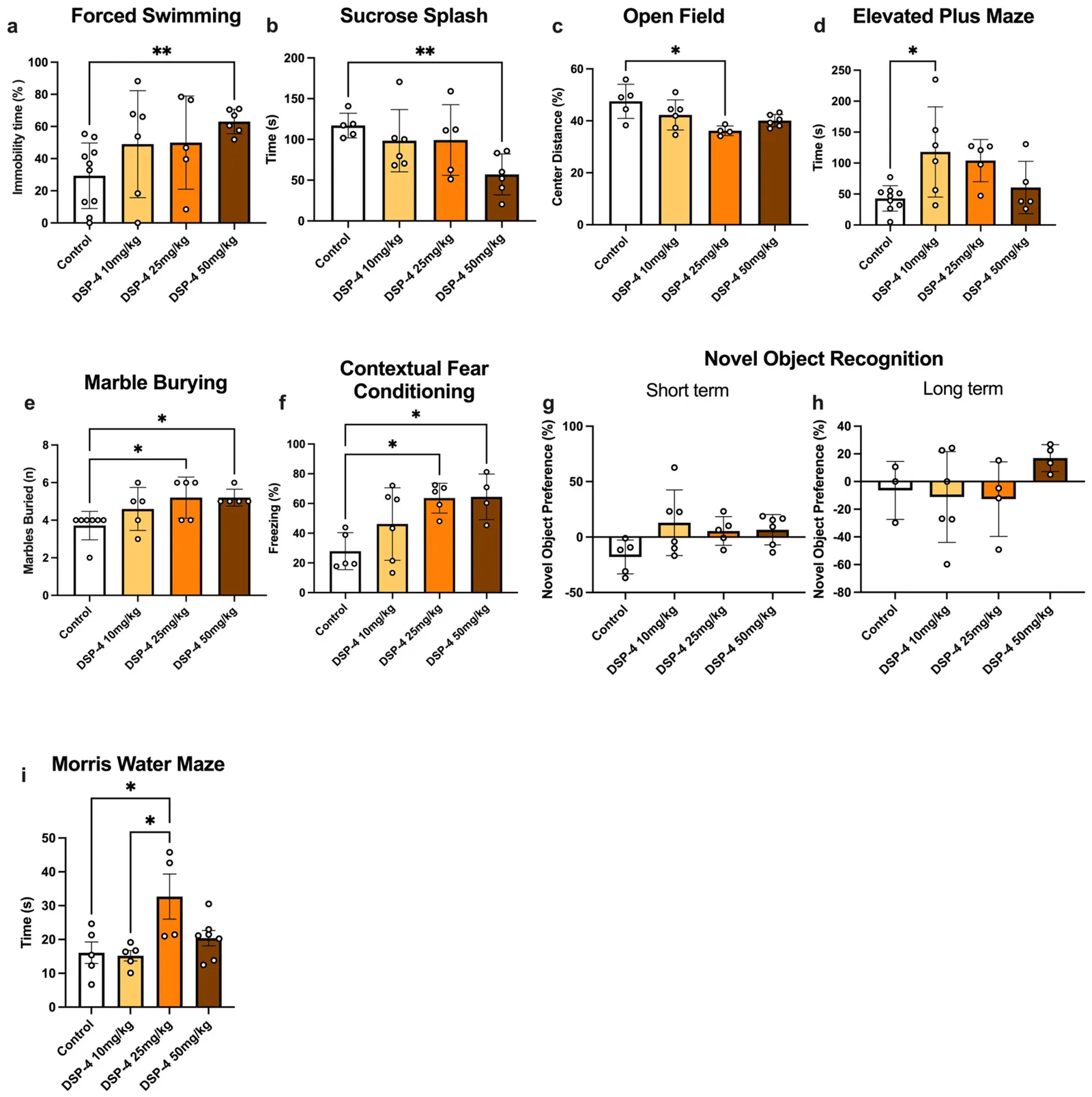
Non-motor characterization of different DSP-4 dosages in DJ-1 Knockout animals. (**a**) Forced Swimming Test to assess depressive-like behavior expressed as a percentage of time spent in immobility. Differences among groups were assessed using Brown–Forsythe and Welch ANOVA tests, revealing a significant effect (Brown–Forsythe: F (3, 12.56) = 2.500, *p* = 0.1070); Welch: W (3, 9.951) = 6.668, *p* = 0.0095). Post hoc comparisons were performed using Dunnett’s T3 multiple comparisons test. (**b**) Sucrose Splash Test to assess depressive-like behavior expressed as the time in seconds (s) mice spent grooming. Differences among groups were assessed using Brown–Forsythe and Welch ANOVA tests, revealing a significant effect (Brown–Forsythe: F (3, 12.35) = 4.485, *p* = 0.0491); Welch: W (3, 9.363) = 6.952, *p* = 0.0094). Post hoc comparisons were performed using Dunnett’s T3 multiple comparisons test. (**c**) Open Field expressed as a percentage of the distance traveled in the center of the arena analyzed with one-way ANOVA (F (3, 17) = 4.586, *p* = 0.0158), followed by Tukey’s multiple comparisons tests. (**d**) Elevated Plus Maze expressed as the time spent in the apparatus’s open arms analyzed with one-way ANOVA (F (3, 21) = 4.297, *p* = 0.0164), followed by Tukey’s multiple comparisons tests. (**e**) Marble Burying to assess anxious-like behavior, expressed as the number of marbles buried, analyzed with one-way ANOVA (F (3, 28) = 3.833, *p* = 0.0277), followed by Tukey’s multiple comparisons tests. (**f**) Contextual Fear Conditioning to assess fear-related memory retrieval, analyzed with one-way ANOVA (F (3, 16) = 4.858, *p* = 0.0137), followed by Tukey’s multiple comparisons tests. (**g**) Novel Object Recognition short-term paradigm to evaluate cognitive deficits. Differences among groups were assessed using Brown–Forsythe and Welch ANOVA tests (Brown–Forsythe: F ((3, 12.05) =2.672, *p* = 0.0945); Welch: W (3, 9.785) = 3.028, *p* = 0.0813). (**h**) Novel Object Recognition long-term paradigm to evaluate cognitive deficits analyzed with one-way ANOVA (F (3, 13) = 1.186, *p* = 0.3534), followed by Tukey’s multiple comparisons tests. (**i**) Morris Water Maze to test spatial recognition expressed as the time in seconds (s) to reach the platform, analyzed with one-way ANOVA (F (3, 17) = 4.715, *p* = 0.0143), followed by Tukey’s multiple comparisons tests. * *p* < 0.05 and ** *p* < 0.01. Data are presented as the mean ± SEM (*n* = 5–10 animals per group).

To assess depressive-like behavior, we selected two behavioral paradigms: The Forced Swimming Test (FST) and the Sucrose Splash Test (SST). FST evaluates helplessness or depressive-like behavior under the concept that rodents show increased periods of inertia when they learn that escape is not possible [19,20]. Our results showed that animals injected with the higher dosage of DSP-4 showed an increased immobility time compared with saline-treated animals, suggesting the presence of a depressive-like behavior (Figure 2a, *p* < 0.01). The SST enhances the need for grooming by splashing animals’ coats with a sucrose solution. When developing depressive-like symptoms, mice usually spend less time grooming and are more immobile [19,20]. Animals treated with the highest dosage spent less time grooming, which is associated with a depressive-like behavior phenotype (Figure 2b, *p* < 0.01).

To assess anxiety-like behavior, the OF and EPM tests make use of mice’s innate tendency to explore new environments and their natural tendency to avoid bright, open places [19]. In the Open Field, animals treated with 25 mg/kg of DSP-4 achieved less distance in the center of the arena, indicating the establishment of anxious-like behavior (Figure 2c, *p* < 0.05). In the Elevated Plus Maze, only animals treated with 10 mg/kg spent more time in the open arms when compared with the control, suggesting that both 25 and 50 mg/kg dosage maintain mice’s natural tendency to avoid open illuminated spaces (Figure 2d, *p* < 0.05). Marble-burying is frequently used to address anxiety related to novelty [19]. When an unfamiliar object is placed in the cage, mice usually start displaying specific behavior, such as burying, indicators of anxious behavior [21]. Our results show that animals treated with either 25 or 50 mg/kg buried more marbles than the control group, frequent in anxious-like-type behavior (Figure 2e, *p* < 0.05).

To address the impact of LC lesion and DJ-1 KO mutation on cognition, we selected behavioral tests to assess spatial memory (Morris Water Maze), learning, and memory (Novel Object Recognition and Contextual Fear Conditioning). Contextual Fear Conditioning (CFC) is used to assess learning and memory formation, usually in relation to fear responses and the formation of fear memories. In CFC, both 25 and 50 mg/kg-treated animals showed an increased freezing response when presented with different stimuli, suggesting an increased fear response (Figure 2f, *p* < 0.05). No differences were found in Novel Object Recognition either in the short- or long-term paradigms (Figure 2g,h). The Morris Water Maze (MWM) is commonly used to evaluate learning and memory, specifically spatial memory acquisition [22]. Distance traveled and latency to reach the platform can be used to evaluate memory learning. Over sessions, healthy animals usually travel shorter distances and require less time to reach the platform, indicating memory retrieval. In the MWM, animals treated with 25 mg/kg DSP-4 took more time to reach the platform compared with controls (Figure 2i, *p* < 0.05), suggesting lower spatial memory retrieval.

Taken together, our results suggest that both the 25 and 50 mg/kg dosages were able to induce the establishment of a non-motor phenotype in the DJ-1 KO animals, consistent with early-onset symptomatology.

### 3.2. Higher-Dose DSP-4 Induces Motor Deficits in DJ-1 Knockout Mice

To address whether noradrenergic lesion could impact motor function in DJ-1 KO animals, we performed commonly used behavioral tests used in current PD models to evaluate gait alterations, motor performance (Motor Swimming Test) and balance (Beam Balance) [23]. This outcome is not only important to understand whether the model has some motor impairments but also to understand whether the results obtained in non-motor tests that require robust motor activity (e.g., Morris Water Maze) could be influenced by any underlying motor alteration.

**Figure 3:**
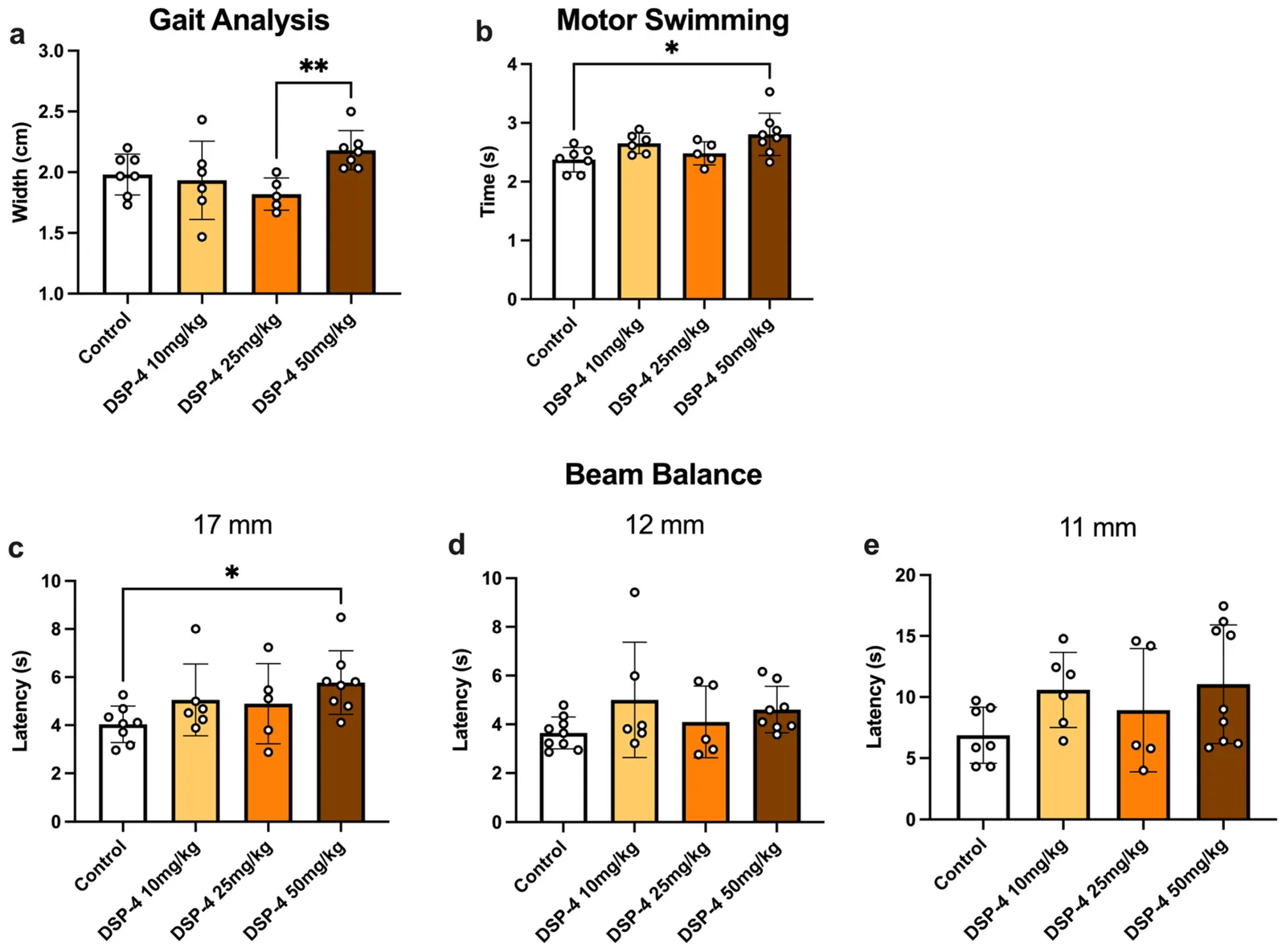
Motor characterization of different DSP-4 dosages in DJ-1 knockout animals. (**a**) Gait Analysis: Differences among groups were assessed using Brown–Forsythe and Welch ANOVA tests, revealing a significant effect (Brown–Forsythe: F (3, 12.22) = 3.163, *p* = 0.0633; Welch: W (3, 11.14) = 5.446, *p* = 0.0150). Post hoc comparisons were performed using Dunnett’s T3 multiple comparisons test. (**b**) Motor Swimming Test showing latency (s) for animals to cross the swimming pool. Differences among groups were assessed using one-way ANOVA (F (3, 22) = 3.908, *p* = 0.0223), followed by Tukey’s multiple comparisons tests. (**c**) Beam Balance (17 mm) showing latency (s) to cross the beam. Differences among groups were assessed using a Kruskal–Wallis test (H = 7.870, *p* = 0.0488), followed by Dunn’s multiple comparisons test. (**d**) Beam Balance (12 mm) showing latency (s) to cross the beam. Data were analyzed using one-way ANOVA (F (3, 24) = 1.335, *p* = 0.2865). (**e**) Beam Balance (11 mm) showing latency (s) to cross the beam. Differences among groups were assessed using a Kruskal–Wallis test (H = 5.429, *p* = 0.1429). * *p* < 0.05 and ** *p* < 0.01 . Data are presented as the mean ± SEM (*n* = 5–10 animals per group).

In the Gait Analysis, animals treated with either dosage failed to differ significantly from the control (Figure 3a). However, animals treated with 50 mg/kg had significantly higher hinder stride width compared with the 25 mg/kg-treated animals, suggesting an enlargement of the stride, consistent with early PD-like alterations (*p* < 0.01). In the Motor Swimming, animals treated with the higher dosage took more time to cross the pool than the controls (Figure 3b, *p* < 0.05), indicating motor deficits. During the Beam Balance, in the round, wider beam (17 mm), the 50 mg/kg-treated animals also showed an increased latency to cross the beam, confirming the establishment of a motor phenotype (Figure 3c, *p* < 0.05).

Taken together, the dose–response characterization revealed a differential effect of DSP-4 on non-motor and motor behaviors. While the 10 and 25 mg/kg doses produced alterations in non-motor behaviors, these effects were not accompanied by significant motor impairments. In contrast, the 50 mg/kg dose induced both non-motor and motor alterations, providing a broader behavioral phenotype relevant to PD. Therefore, we selected the 50 mg/kg DSP-4 dose for subsequent experiments, as it produced the most consistent combination of motor and non-motor alterations while not significantly compromising animal welfare, thus providing the most suitable dosage to induce both non-motor and motor PD-relevant phenotypes.

### 3.3. Noradrenergic Depletion Induces Anxiety-, Depression-, and Fear-Related Behaviors in DJ-1 KO Mice

Due to the limited characterization of non-motor alterations in the DJ-1 KO model, we investigated the effects of DSP-4 treatment in this background, while also assessing the independent contributions of noradrenergic lesioning and the DJ-1 mutation.

**Figure 4:**
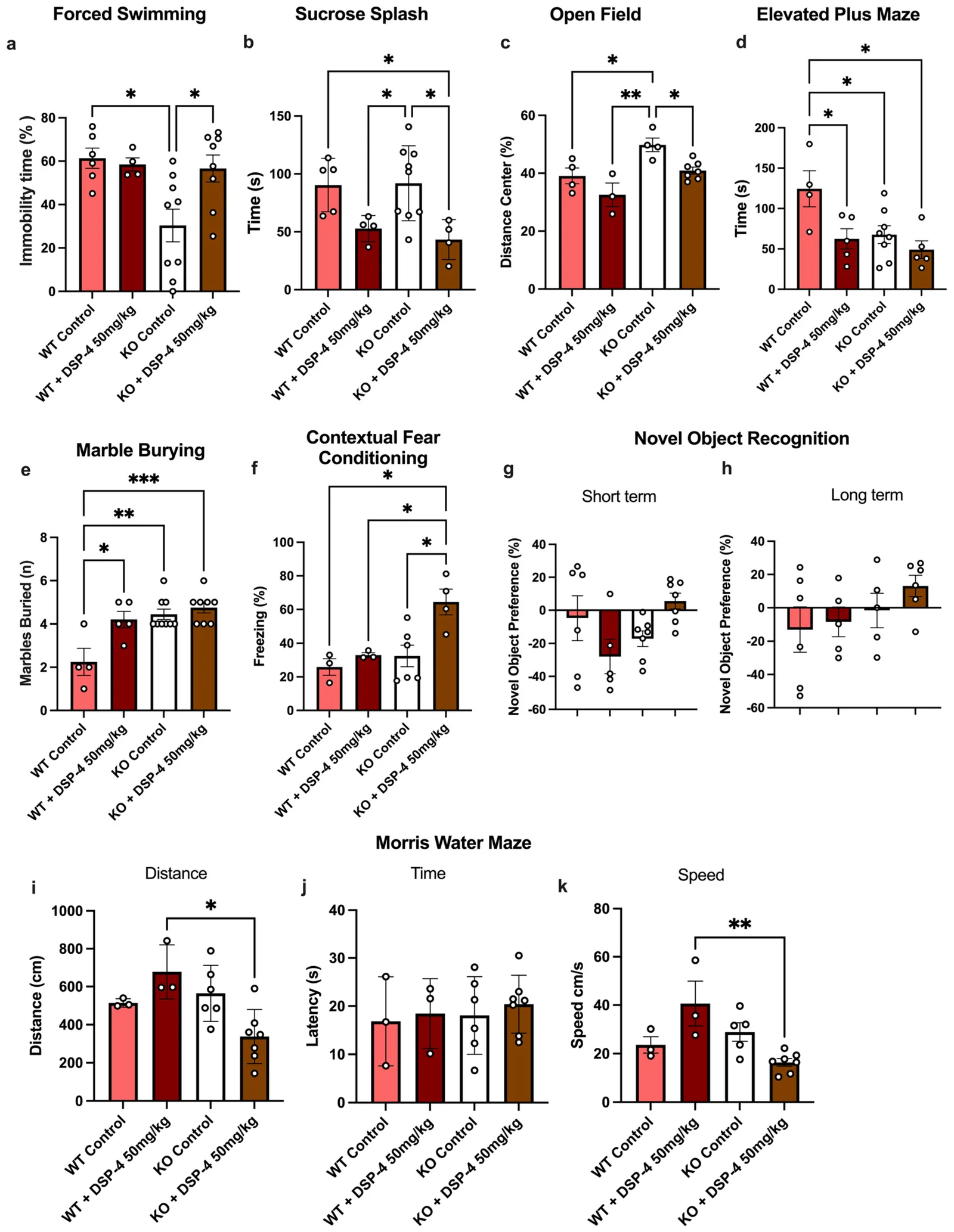
DSP-4 elicits non-motor deficits in DJ-1 knockout animals. (**a**) Forced Swimming Test to assess depressive-like behavior expressed as a percentage of the time spent in immobility analyzed with one-way ANOVA (F (3, 23) = 5.283, *p* = 0.0064), followed by Tukey’s multiple comparisons tests. (**b**) Sucrose Splash Test to assess depressive-like behavior, expressed as the time, in seconds (s), mice spent grooming. Differences among groups were assessed using Brown–Forsythe and Welch ANOVA tests, revealing a significant effect (Brown–Forsythe: F (3, 16.71) =7.078, *p* = 0.0028); Welch: W (3, 8.875) = 6.563, *p* = 0.0124). Post hoc comparisons were performed using Dunnett’s T3 multiple comparisons test. (**c**) Open Field expressed as a percentage of the distance traveled in the center of the arena analyzed with one-way ANOVA (F (3, 14) = 8.022, *p* = 0.0024), followed by Tukey’s multiple comparisons tests. (**d**) Elevated Plus Maze expressed as the time spent in the apparatus’s open arms analyzed with one-way ANOVA (F (3, 18) = 4.679, *p* = 0.0138), followed by Tukey’s multiple comparisons tests. (**e**) Marble Burying to assess anxious-like behavior, expressed as the number of marbles buried, analyzed with one-way ANOVA (F (3, 22) = 8.697, *p* = 0.0005), followed by Tukey’s multiple comparisons tests. (**f**) Contextual Fear Conditioning to assess fear-related memory retrieval analyzed with one-way ANOVA (F (3, 12) = 6.615, *p* = 0.0069), followed by Tukey’s multiple comparisons tests. (**g**) Novel Object Recognition short-term paradigm to evaluate cognitive deficits. Differences among groups were assessed using a Kruskal–Wallis test (H = 6.842, *p* = 0.0771). (**h**) Novel Object Recognition long-term paradigm to evaluate cognitive deficits. Differences among groups were assessed using a Kruskal–Wallis test (H = 3.782, *p* = 0.2860). (**i**) Morris Water Maze test to assess spatial recognition, expressed as the distance, in centimeters (cm), to reach the platform. Differences among groups were assessed using a Kruskal–Wallis test (H = 10.16, *p* = 0.0055). Pos hoc comparisons using Dunn’s correction. (**j**) Morris Water Maze test to assess spatial recognition, expressed as time, in seconds (s), to reach the platform. Differences among groups were assessed using a Kruskal–Wallis test (H = 0.2654, *p* = 0.9721). (**k**) Morris Water Maze test to assess spatial recognition, expressed as the speed (cm/s) achieved during the test, analyzed with one-way ANOVA (F (3, 14) = 6.405, *p* = 0.0059), followed by Tukey’s multiple comparisons tests. * *p* < 0.05, ** *p* < 0.01 and *** *p* < 0.001. Data are presented as the mean ± SEM (*n* = 3–10 animals per group). Abbreviations: wild type (WT) and knockout (KO).

In the FST, DJ-1 KO animals spent less time in immobility when compared with the WT control animals (Figure 4a, *p* < 0.05). However, the DJ-1 KO DSP-4-treated animals showed an increased immobility time when compared with the DJ-1 KO control animals (*p* < 0.05) but not when compared with the WT controls. This suggests that the DJ-1 KO mutation leads to a reduced depressive-like behavior, which is reversed by the administration of DSP-4. In the SST, no differences were found between the WT and DJ-1 KO control animals (Figure 4b). However, both the DJ-1 KO DSP-4- and WT DSP-4-treated animals showed decreased grooming activity compared with the DJ-1 KO control animals (*p* < 0.05). This suggests that DSP-4 lesion induces a depressive-like phenotype in both WT and DJ-1 Ko animals. However, only the DJ-1 KO DSP-4-treated animals showed a significant reduction in grooming activity when compared with the WT control (*p* < 0.05). In the OF, the DJ-1 KO control animals spent more time in the center of the arena when compared with the WT control animals, suggesting that the DJ-1 KO animals were less anxious than the WT animals (Figure 4c, *p* < 0.05). However, the DJ-1 KO DSP-4-treated animals showed a decreased activity in the center, suggesting a more anxious-like phenotype when compared with the DJ-1 KO control animals (*p* < 0.05). In the EPM, the WT DSP-4, DJ-1 KO and DJ-1KO DSP-4 animals showed a reduction in the time spent in the open arms when compared with the WT control group (Figure 4d, *p* < 0.05). This suggests an anxious-like behavior in the DJ-1 KO animals and reinforces the results from the previous task, showing that the combination of LC lesioning in the DJ-1 KO animals results in anxious-like behavior. In the Marble Burying test, the WT DSP-4-treated, DJ-1 KO control and DJ-1 KO DSP-4-treated animals showed increased burying activity when compared with the WT controls (Figure 4e, *p* < 0.05, *p* < 0.01 and *p* < 0.001, respectively). This indicates that the DJ-1 KO animals are more sensitive to novel stimuli and suggests that the DSP-4 lesioning of the LC also results in an anxious-like behavior. However, the DJ-1 KO DSP-4-treated animals did not show differences compared with the DJ-1 KO control animals, suggesting that the LC lesion does not aggravate this phenotype. Overall, in all three behavior tasks, the DJ-1 KO DSP-4-treated animals showed consistent anxious-like behaviorial responses.

In the CFC, on the traumatic context setup, the DJ-1 KO DSP-4-treated animals showed an increased freezing response when compared with the DJ-1 KO control, WT and WT DSP-4-treated animals (Figure 4f, *p* < 0.05). This suggests that noradrenergic lesioning in DJ-1 KO animals results in a heightened vigilance state and fear-memory retrieval. Moreover, our results suggest that the administration of DSP-4 alone is not sufficient to affect the retrieval of fear memory, as no differences were found between the WT control and the WT DSP-4-treated animals. The NOR test is often used to assess short- and long-term memory retrieval in animals without the presence of an aversive stimulus. However, the WT vehicle group did not discriminate against the objects used, therefore invalidating the interpretation of the results (Figure 4g,h). In the MWM, the DJ-1 KO DSP-4-treated animals traveled shorter distances to encounter the platform when compared with the WT DSP-4-treated animals, suggesting better memory acquisition (Figure 4i, *p* < 0.05). Additionally, the average speed that the animals achieved during the test was also used as an additional measure of motor function (Figure 4k). When compared with the WT DSP-5 treated animals, the DJ-1 KO DSP-4-treated animals reached a lower speed, indicating motor deficits, which is aligned with our previous results suggesting that a DSP-4 lesion in DJ-1 KO animals results in motor deficits (*p* < 0.01).

These findings indicate that the combination of a DJ-1 deficiency and DSP-4-induced noradrenergic degeneration is associated with a distinct non-motor behavioral profile. The DJ-1 KO mice exhibited alterations in baseline anxiety- and depressive-like behaviors, while DSP-4 treatment was associated with increased anxiety-like responses and altered grooming and immobility behaviors. Notably, the DJ-1 KO mice receiving DSP-4 consistently displayed anxiety-like behaviors across the Open Field, Elevated Plus Maze, and Marble Burying tests, alongside an enhanced freezing response during Contextual Fear Conditioning. In the Morris Water Maze, these animals showed reduced platform acquisition distance but also lower swimming speed, highlighting the need to consider motor performance when interpreting spatial learning outcomes. Overall, the results suggest that the interaction between a DJ-1 deficiency and noradrenergic degeneration affects multiple behavioral domains, particularly anxiety-like behavior and contextual fear responses, while the effects on depressive-like behavior and memory remain more complex.

### 3.4. Noradrenergic Degeneration Exacerbates Motor Dysfunction in DJ-1 Knockout Mice

To determine whether DJ-1 deficiency and selective noradrenergic degeneration contribute to motor dysfunction, we further assessed motor performance in the DJ-1 KO and WT mice in the presence or absence of DSP-4-induced LC degeneration. Motor dysfunction is a defining clinical feature of PD and is closely associated with nigrostriatal dopaminergic dysfunction. Therefore, characterization of motor performance is an important component when evaluating the face validity of experimental PD models.

**Figure 5:**
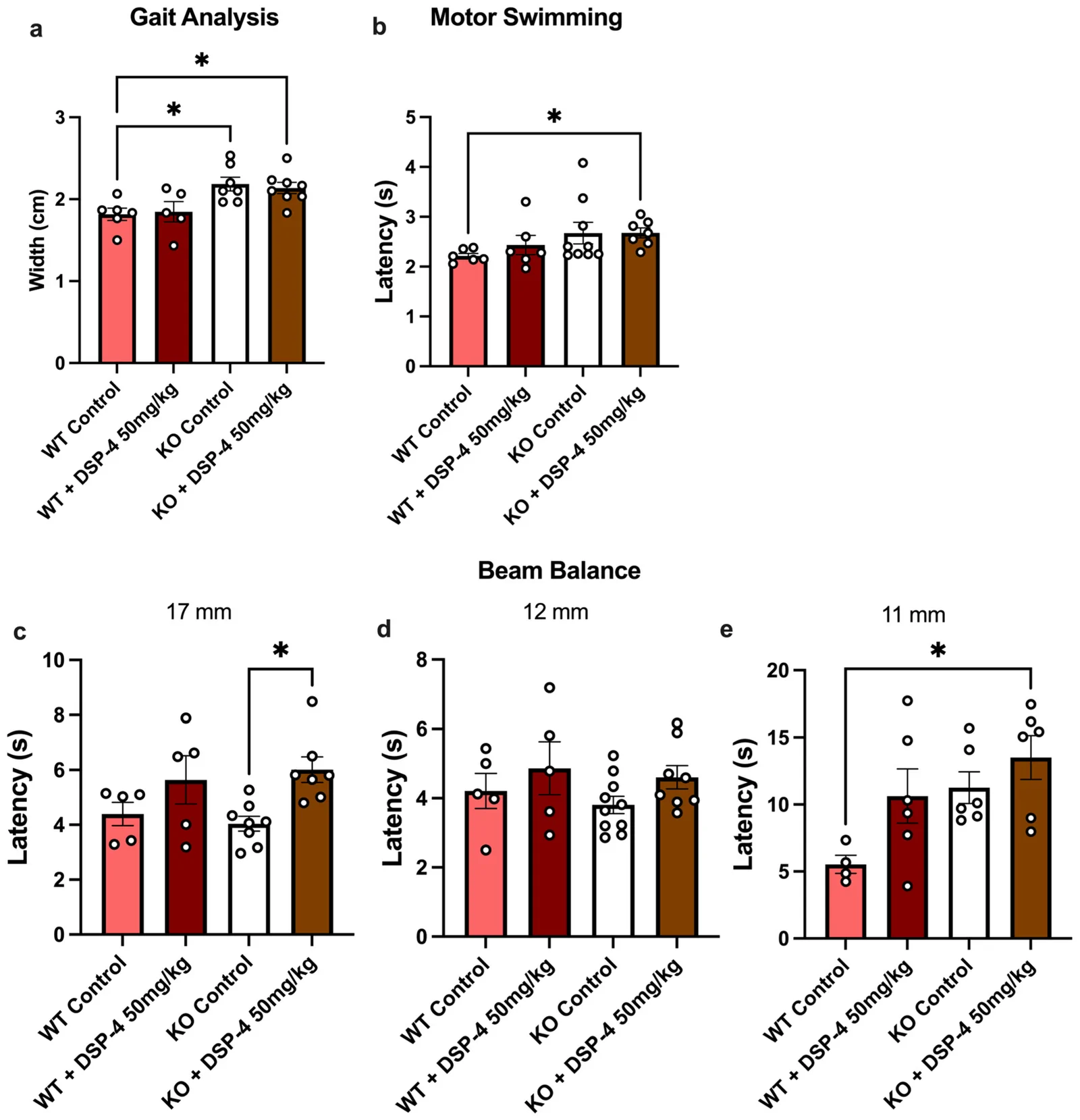
Noradrenergic lesion elicits motor deficits in DJ-1 KO animals. (**a**) Gait Analysis: Differences among groups were assessed using Brown–Forsythe and Welch ANOVA tests, revealing a significant effect (Brown–Forsythe: F (3, 15.92) = 4.675, *p* = 0.0158; Welch: W (3, 11.02) = 4.792, *p* = 0.0226). Post hoc comparisons were performed using Dunn’s multiple comparisons test. (**b**) Motor Swimming Test showing latency (s) for animals to cross the swimming pool. Differences among groups were assessed using a Kruskal–Wallis test (H = 8.644, *p* = 0.0344). Post hoc comparisons were performed using Dunnett’s T3 multiple comparisons test. (**c**) Beam Balance (17 mm) showing latency (s) to cross the beam. Differences among groups were analyzed using one-way ANOVA (F (3, 21) = 4.00, *p* = 0.0213), followed by Tukey’s multiple comparisons test. (**d**) Beam Balance (12 mm) showing latency (s) to cross the beam. Data were analyzed using one-way ANOVA (F (3, 24) = 1.342, *p* = 0.2843). (**e**) Beam Balance (11 mm) showing latency (s) to cross the beam. Differences among groups were assessed using a Kruskal–Wallis test (H = 8.343, *p* = 0.0394), followed by Dunn’s multiple comparisons test. * *p* < 0.05. Data are presented as the mean ± SEM (*n* = 4–10 animals per group). Abbreviations: wild type (WT) and knockout (KO).

In the Gait Analysis, both the DJ-1 control and DJ-1 DSP-4-treated animals showed an increased base width when compared with the WT control animals (Figure 5a, *p* < 0.05). In the Motor Swimming Test, used to assess fine motor coordination, only the DSP-4-treated DJ-1 KO animals showed a significant increase in the time to reach the platform compared with the WT control animals (Figure 5b, *p* < 0.05). In the Beam Balance, the animals were challenged with three different beams. In the 17 mm beam, the DJ-1 KO DSP-4-treated animals took more time to cross the beam when compared with the DJ-1 KO control animals (Figure 5c, *p* < 0.05). No differences were found among groups in the 12 mm beam (Figure 5d). In the 11 mm beam, only the DJ-1 KO DSP-4-treated animals showed increased latency to cross the beam when compared with the WT control animals (Figure 5e).

Our results suggest that the DJ-1 KO animals present a mild motor alteration but, the combination of the LC lesion with the DJ-1 KO mutation results in a pronounced motor phenotype. In previous work, when combining a 6-OHDA lesion in the nigrostriatal pathway with the LC lesioning, the combined lesion did not result in altered motor behavior [24]. This suggests that the effect observed is likely due to the interaction of the noradrenergic lesioning with the altered pathways in the DJ-1 mutation, leading to the establishment of motor symptoms.

### 3.5. Noradrenergic Lesioning Aggravates Dopaminergic Loss in the Striatum of DJ-1 KO Mice

**Figure 6:**
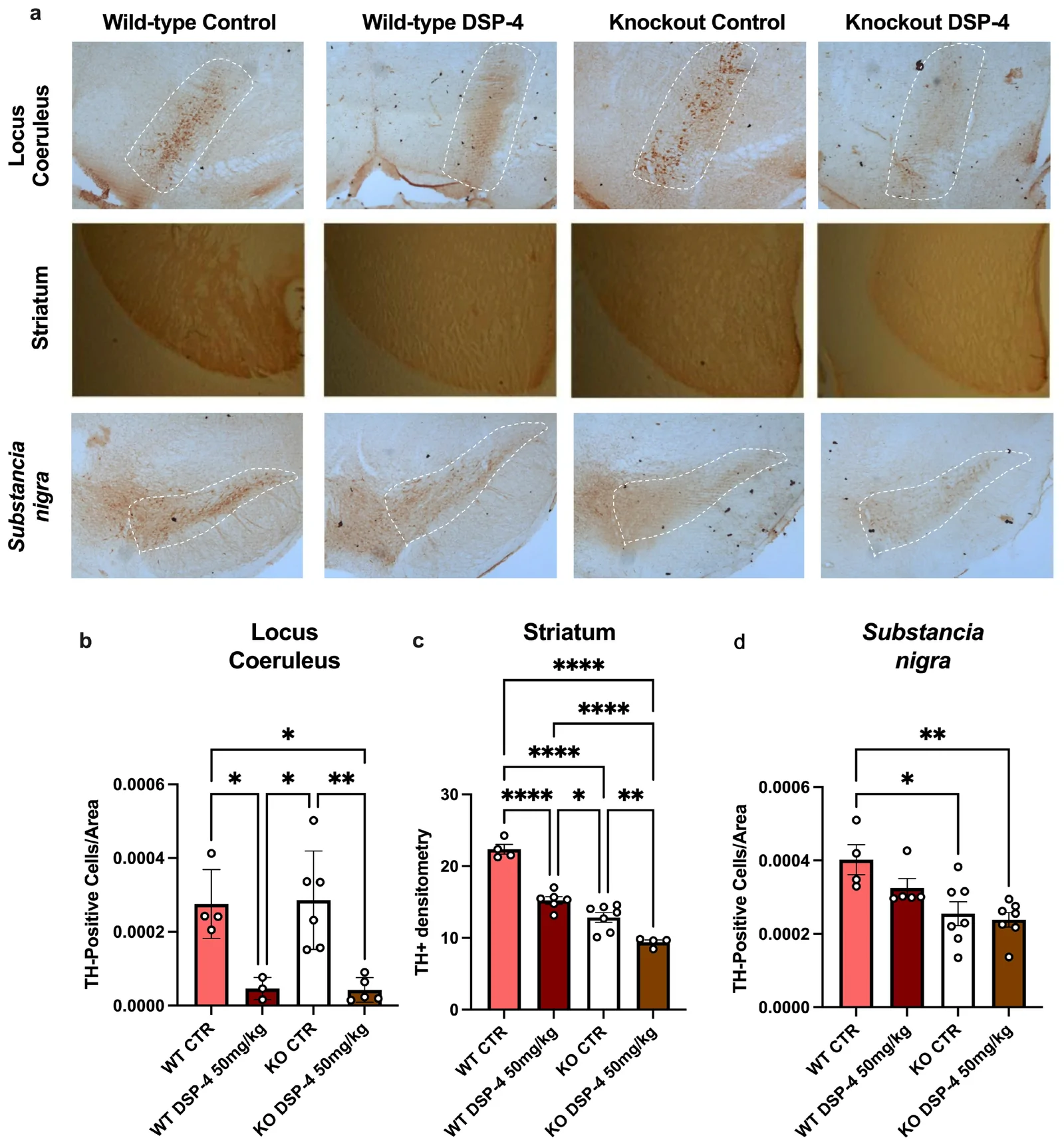
Noradrenergic lesion worsens nigrostriatal loss. (**a**) Representative images of locus coeruleus (LC), striatum and Substancia nigra (SN). (**b**) Quantification of positive Tyrosine hydroxylase (TH) cells in the locus coeruleus (LC), expressed as TH-positive cells per area analyzed with one-way ANOVA (F (3,14) = F (3, 14) = 9.657, *p* < 0.001). Post hoc comparisons were performed using Tukey’s correction. (**c**) Quantification of TH densitometry of the striatum analyzed with one-way ANOVA F (3, 17) = 62,13, *p* < 0.0001. Post hoc comparisons were performed using Tukey’s correction. (**d**) Quantification of positive TH cells counts in the substancia nigra pars compacta (SNpc) expressed as TH-positive cells per area analyzed with one-way ANOVA F (3, 19) = 5637, *p* = 0.0062. Post hoc comparisons were performed using Tukey’s correction. * *p* < 0.05, ** *p* < 0.01 and **** *p* < 0.0001. Data are presented as the mean ± SEM (*n* = 5–10 animals per group). Abbreviations: wild type (WT) and knockout (KO).

To understand whether the readouts obtained in the behavioral analysis are related to noradrenergic and/or nigrostriatal degeneration, we performed a histological analysis. Given the high selectivity of DSP-4 for LC neurons, we started by evaluating LC degeneration (Figure 6a).

Our results showed a decrease of ≈80% LC neurons between the WT DSP-4 animals and WT vehicle group (Figure 6a, *p* < 0.05) and a decrease of ≈85% LC neurons between the DJ-1 KO DSP-4 and DJ-1 KO vehicle groups (Figure 6a, *p* < 0.05). Despite previous findings reporting that aged DJ-1 KO animals present LC degeneration [25], no differences were found between the WT and DJ-1 KO vehicle groups or between the WT DSP-4 and DJ-1 KO DSP-4 groups. Herein, our results suggest that the DJ-1 mutation does not seem to trigger noradrenergic degeneration in the LC, nor increase the vulnerability to DSP-4’s effects.

The loss of dopaminergic neurons in the nigrostriatal pathway is one of the main pathological features of PD. Loss of TH+ cells in the SNpc and a decrease in TH+ densitometry in the striatum are usually observed in most PD models. To evaluate the effects of DJ-1 KO mutation and the effects of DSP-4-induced NA depletion, we performed IHC on the SNpc and the STR (Figure 6a). In the striatum, the WT DSP-4-treated animals showed reduced TH densitometry compared with the WT control animals (Figure 6b, *p* < 0.001). The DJ-1 KO control animals also showed a reduction in TH densitometry compared with the WT controls (*p* < 0.001). While this is in agreement with some studies [25], others reported that aged DJ-1 KO animals do not present alterations in dopaminergic neuron content or dopamine turnover [26]. Interestingly, the DJ-1 KO DSP-4-treated animals showed a lower TH densitometry, with it being significantly decreased compared with the DJ-1 KO control (*p* < 0.01), WT DSP-4-treated (*p* < 0.001) and WT control animals (*p* < 0.001). Importantly, in the SNpc, both the DJ-1 KO control (*p* < 0.05) and DJ-1 KO DSP-4-treated animals (Figure 6c, *p* < 0.01) showed a significant reduction in the number of TH+ cells in the SNpc compared with the WT control animals. These results suggest that while lesioning, the LC seems to induce striatal dopaminergic loss; this aggravates the degeneration evoked by the DJ-1 KO mutation, which might explain the previous observed motor deficits.

## 4. Discussion

In the present study, we demonstrate that selective noradrenergic degeneration of the locus coeruleus significantly aggravates dopaminergic vulnerability and promotes both motor and non-motor impairments in DJ-1 KO mice. By combining a genetic susceptibility model with DSP-4–induced LC lesioning, we established a mouse model that recapitulates key features associated with early-stage PD. Importantly, our findings support the concept that noradrenergic degeneration functions as a disease modifier in a genetically vulnerable background rather than representing a secondary or independent pathological event.

Given the increased susceptibility of DJ-1 KO animals to other toxins (e.g., 6-OHDA and MPTP) described in several studies, we started our study by performing a dose-dependant assessment of DSP-4 on DJ-1 KO animals [15,27]. Previous studies have shown that lower dosages (10 and 25 mg/kg) produce a partial lesion, without an effect on behavior, while higher dosages (50 mg/kg) have a greater impact and are able to directly affect motor behavior [28,29,30,31,32,33]. Our results showed that both 10 and 25 mg/kg DSP-4 dosages produced alterations in non-motor behaviors, specifically in anxious- and depressive-like behaviors. However, these effects were not accompanied by significant motor impairments, which is still a critical requirement for PD modeling. Nevertheless, the 50 mg/kg dosage was able to induce non-motor alterations in both anxious- and depressive-like behaviors, as well as cognitive performance, while also affecting motor conditioning. After establishing 50 mg/kg as the ideal dosage, we wanted to characterize the effect of both the DJ-1 mutation and the DSP-4 lesion in non-motor alterations, which frequently precede classical motor manifestations in PD and are strongly associated with early LC pathology.

Our results showed that DSP-4 administration produced a consistent anxiety- and depressive-like phenotype, particularly in DJ-1 KO animals. Some studies have linked increased LC activity and the development of anxiety-related disorders. In the context of depression, some studies showed a correlation between the decline of NA content and the reduction in LC pigmentation with the prevalence of depression in PD patients [3,6,7,32]. Increased immobility in the Forced Swimming Test and reduced grooming behavior in the Sucrose Splash Test indicate the emergence of depressive-like features following noradrenergic depletion. In studies where NA was depleted, mice showed an increased immobility time in the FST compared with control animals [30,32,34]. Similarly, alterations observed in the Open Field, Elevated Plus Maze, and Marble Burying tasks support the development of anxiety-like behavior. Some authors reported that LC-lesioned rats had reduced anxious behavior [35]. However, other studies showed that in mice, NA depletion alone is not sufficient to significantly affect the exploration of open contexts [32]. Notably, DJ-1 KO animals alone displayed reduced immobility and increased center exploration, suggesting altered baseline stress coping or anxiety-related behavior. These findings are aligned with previous studies, showing that combining LC and nigrostriatal lesions results in increased anxious phenotype [24]. This phenotype may reflect compensatory monoaminergic adaptations in the absence of DJ-1, potentially involving altered noradrenergic tone. The reversal of this phenotype after DSP-4 administration suggests that noradrenergic signaling modulates behavior in this model. These observations are consistent with clinical data linking LC depigmentation and reduced noradrenaline levels to depression and anxiety in PD patients [3,4,7,16,17].

In the Contextual Fear Conditioning task, only the DJ-1 KO animals subjected to LC lesioning exhibited enhanced freezing responses, suggesting heightened fear memory retrieval or increased vigilance. Given the well-established role of LC projections to the amygdala and hippocampus in regulating arousal and threat-related learning, these findings indicate that noradrenergic disruption in a genetically susceptible background dysregulates limbic circuitry [5,16,17,18,32,36,37]. Importantly, DSP-4 treatment alone did not alter fear responses in wild-type animals, reinforcing the hypothesis that genetic vulnerability is required for the full behavioral phenotype to emerge. Together, these data suggest that noradrenergic degeneration interacts with DJ-1 deficiency to unmask or exacerbate non-motor symptomatology relevant to early PD.

Since motor phenotypes are a key feature of PD models, it is important to highlight that DJ-1 KO mice treated with DSP-4 exhibited deficits in Motor Swimming and Beam Balance tasks, along with gait alterations characterized by increased base width. These findings are consistent with previous studies showing that LC lesioning results in aggravated motor deficits [38,39]. The most consistent impairments were observed in tasks requiring fine motor coordination and balance, which are sensitive to early nigrostriatal dysfunction (e.g., Motor Swimming and Beam Balance tests). This feature enhances the translational relevance of the model, as early-stage PD patients often present with subtle motor abnormalities before extensive SNpc degeneration becomes evident. We observed that DJ-1 KO animals have an increased stride width when compared with control, a gait dysfunction typical in PD patients and which has been previously described in a DJ-1 KO model, but it does not seem to be aggravated or induced by DSP-4 lesioning [15,25]. Importantly, the overall behavioral phenotype remained moderate and did not compromise general welfare, supporting the utility of this model for studying early pathological processes and therapeutic interventions while still allowing to gain insight into motor performance.

We observed a decrease of 85% in the content of TH-positive neurons in the LC of the DSP-4-treated WT and KO mice, confirming the effectiveness of the lesion. In rats, when administered the same DSP-4 dosage as we used in this study (50 mg/kg), a decrease of 70% in DBH-positive cells in the LC was observed [29,30,31,32,33,34,37,38,39]. Other studies conducted in mice only reported a 34% decrease in DBH-positive cells in the LC when compared with WT animals [28,29,30,31,32,33,34,38,39]. Yet some studies in both mice and rats showed that DSP-4 induces a decrease or complete depletion in the concentration of NA but does not lead to neuronal loss [33]. Our results suggest that DSP-4 exerts a significant impact on LC, leading to substantial neuronal depletion. Moreover, a significant finding of this study is that LC degeneration significantly worsens striatal dopaminergic deficits in DJ-1 KO mice. While DJ-1 deficiency alone resulted in reduced TH-positive cell counts in the SNpc and in the striatum, DSP-4 only led to a pronounced reduction in striatal TH densitometry, exceeding the effects observed in either condition alone. Interestingly, LC lesioning did not further reduce SNpc neuronal counts but predominantly affected dopaminergic terminals within the striatum of DJ-1 KO animals. This pattern suggests a retrograde degenerative process, consistent with early PD pathology in which striatal denervation precedes extensive nigral neuronal loss, which later results in motor alterations [1,2]. These findings support a previous hypothesis that noradrenergic projections modulate dopaminergic terminals and that noradrenalin loss increases dopaminergic neurons’ vulnerability to degeneration [17,18,36]. While we were not able to address the specific mechanisms underlying these effects, several factors may account for this interaction. Noradrenaline modulates dopaminergic transmission, regulates neuroinflammatory responses, and contributes to oxidative stress buffering [17,18,36]. DJ-1, in turn, plays a critical role in mitochondrial homeostasis and protection against oxidative stress [12,13,14]. The combination of impaired anti-oxidant defense due to DJ-1 deficiency and reduced noradrenergic support may compromise dopaminergic terminal integrity, thereby amplifying striatal degeneration. Additionally, alterations in extracellular dopamine turnover following DSP-4 treatment may further destabilize dopaminergic signaling. Although the precise molecular mechanisms remain to be determined, the present data provide experimental evidence for a functional interaction between the LC–noradrenergic system and the nigrostriatal pathway. Future studies incorporating direct neurochemical measurements of noradrenaline and dopamine levels, as well as assessments of oxidative stress and inflammatory markers, would further clarify the mechanisms underlying the observed interactions. Nevertheless, although DSP-4 is widely used to selectively lesion LC neurons, off-target or systemic effects cannot be entirely excluded [34,40]. Moreover, some behavioral paradigms rely on intact motor performance, which may confound interpretation of cognitive outcomes, as suggested by reduced swimming speed in the Morris Water Maze paradigm.

Beyond demonstrating the behavioral and neuropathological consequences of combining DJ-1 deficiency with selective noradrenergic degeneration, the present study provides a framework for investigating how distinct pathological processes interact to shape neuronal vulnerability in PD. A key contribution of this work is the identification of a differential impact of LC degeneration on the nigrostriatal system, with a pronounced effect on striatal dopaminergic terminals in the absence of a corresponding exacerbation of SNc neuronal loss. This observation supports the concept that dopaminergic dysfunction may emerge at different anatomical levels depending on the underlying pathological context and highlights the importance of considering axonal and terminal integrity, rather than neuronal cell loss alone, when evaluating PD progression. Furthermore, the emergence of motor and non-motor abnormalities following the combination of genetic susceptibility and noradrenergic degeneration provides an experimental framework in which interactions between the LC and dopaminergic systems can be investigated across multiple behavioral paradigms. More broadly, this model may facilitate the study of mechanisms linking early multisystem dysfunction to later nigrostriatal pathology and can be used to evaluate therapeutic approaches aimed at preserving noradrenergic and dopaminergic circuit function. Thus, the present findings extend current experimental approaches to PD by incorporating the interaction between genetic vulnerability and degeneration of a non-dopaminergic system, providing a more integrated perspective on the mechanisms underlying disease heterogeneity and progression.

## 5. Conclusions

**Figure 7:**
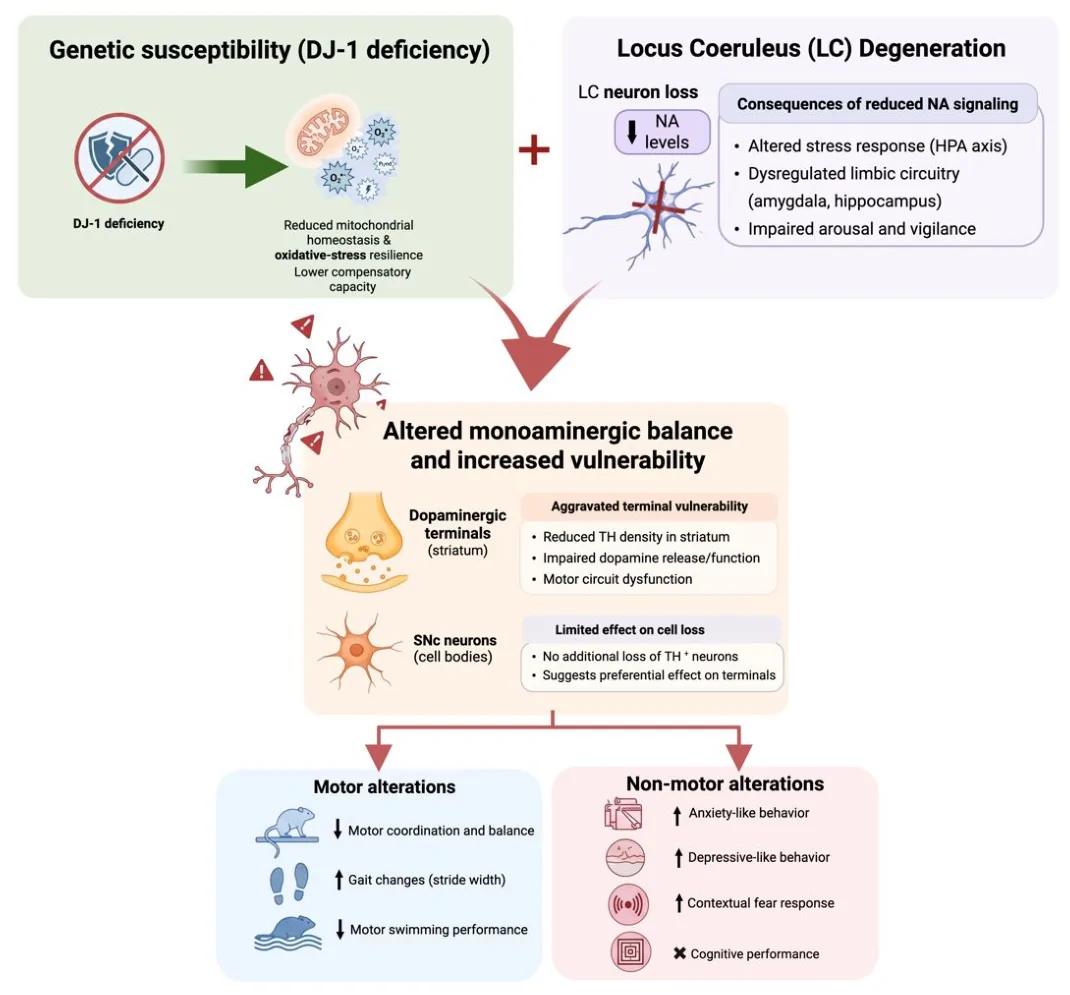
Proposed mechanistic model linking DJ-1 deficiency and locus coeruleus degeneration to dopaminergic vulnerability and behavioral alterations. Schematic representation of the proposed interaction between genetic susceptibility caused by DJ-1 deficiency and DSP-4-induced locus coeruleus (LC) degeneration. DJ-1 deficiency is proposed to compromise mitochondrial homeostasis and oxidative-stress resilience, thereby reducing cellular compensatory capacity and increasing susceptibility to additional pathological stress. LC degeneration results in substantial noradrenergic neuronal loss and reduced noradrenaline (NA) signaling, which may alter stress responses, arousal and vigilance, and limbic circuitry involving the amygdala and hippocampus. The convergence of these pathological processes is proposed to produce an altered monoaminergic balance and increased vulnerability of the nigrostriatal system. In particular, the combined condition was associated with a pronounced reduction in striatal tyrosine hydroxylase (TH) density and, therefore, is proposed to preferentially affect dopaminergic terminal integrity and function, whereas no additional significant loss of TH-positive substantia nigra pars compacta (SNc) neuronal cell bodies was observed. This differential vulnerability may contribute to impaired dopaminergic signaling and motor circuit dysfunction. In parallel, disruption of LC noradrenergic signaling may contribute to alterations in limbic and stress-related circuits, resulting in increased anxiety-like and depressive-like behaviors and enhanced contextual fear responses. Cognitive performance showed more complex effects and was influenced by motor performance in some paradigms. Collectively, the interaction between DJ-1 deficiency and noradrenergic degeneration is proposed to produce a broader motor and non-motor phenotype through the combined effects on dopaminergic terminals and limbic circuitry. The mechanistic relationships depicted are based on the present findings and previously reported evidence and represent a working model rather than directly demonstrated molecular mechanisms (created with BioRender.com).

In summary, our study demonstrates that selective noradrenergic degeneration significantly aggravates nigrostriatal dopaminergic vulnerability in DJ-1 knockout. Furthermore, the combination of DJ-1 deficiency and DSP-4-induced LC degeneration was associated with a broader non-motor phenotype, including increased anxious- and depressive-like behaviors and enhanced contextual fear responses, together with impairments in motor coordination and balance. These findings are particularly relevant given the involvement of the LC–noradrenergic system in the regulation of mood, arousal, stress responses, and fear-related behaviors and support the notion that LC degeneration may contribute not only to motor dysfunction through its impact on dopaminergic terminals but also to the emergence or aggravation of non-motor manifestations associated with PD (Figure 7). The emergence of these alterations in the combined model further suggests that genetic susceptibility and noradrenergic dysfunction may interact to shape the expression and severity of non-motor phenotypes.

In parallel, the combined DJ-1 KO/DSP-4 condition was associated with impairments in motor coordination and balance and with a pronounced reduction in striatal TH density. The absence of additional significant loss of SNpc TH-positive neurons suggests that noradrenergic degeneration may preferentially affect dopaminergic terminals at this stage of the model. Together, these behavioral and neuropathological findings highlight LC degeneration as a relevant contributor to the broader phenotypic consequences of DJ-1 deficiency, extending beyond nigrostriatal motor dysfunction to encompass non-motor alterations (Figure 7). The combined DJ-1 KO/DSP-4 model, therefore, provides a useful experimental framework for investigating the interplay between noradrenergic and dopaminergic systems and for exploring mechanisms and therapeutic strategies targeting the locus coeruleus–nigrostriatal axis.

## Data Availability

The original contributions presented in this study are included in the article/Supplementary Materials. Further inquiries can be directed to the corresponding authors.

## Supplementary Materials

The following supporting information can be downloaded at https://www.mdpi.com/article/10.3390/cimb48101047/s1.

## Abbreviations

The following abbreviations are used in this manuscript:

CFC	Contextual Fear Conditioning
EPM	Elevated Plus Maze
FST	Forced Swimming Test
IT	Immobility Time
i.p.	Intraperitoneal Injection
KO	Knockout
LC	Locus Coeruleus
MWM	Morris Water Maze
DSP-4	N-(2-Chloroethyl)-N-Ethyl-2-Bromobenzylamine
NOR	Novel Object Recognition
PD	Parkinson’s Disease
SNpc	Substancia nigra pars compacta
SST	Sucrose Splash Test
TH	Tyrosine Hydroxylase
WT	Wild Type
