# Supplementary material for: Modeling Parkinson’s Disease Through Combined DJ-1 Deficiency and Noradrenergic Degeneration
Source: Curr Issues Mol Biol. 2026 Oct 10;48(10):1047. doi: 10.3390/cimb48101047 (PMC13703393; doi:10.3390/cimb48101047)
Supplement: Supplementary file 1 [file cimb-48-01047-s001.zip › cimb-4588534-supplementary.pdf]

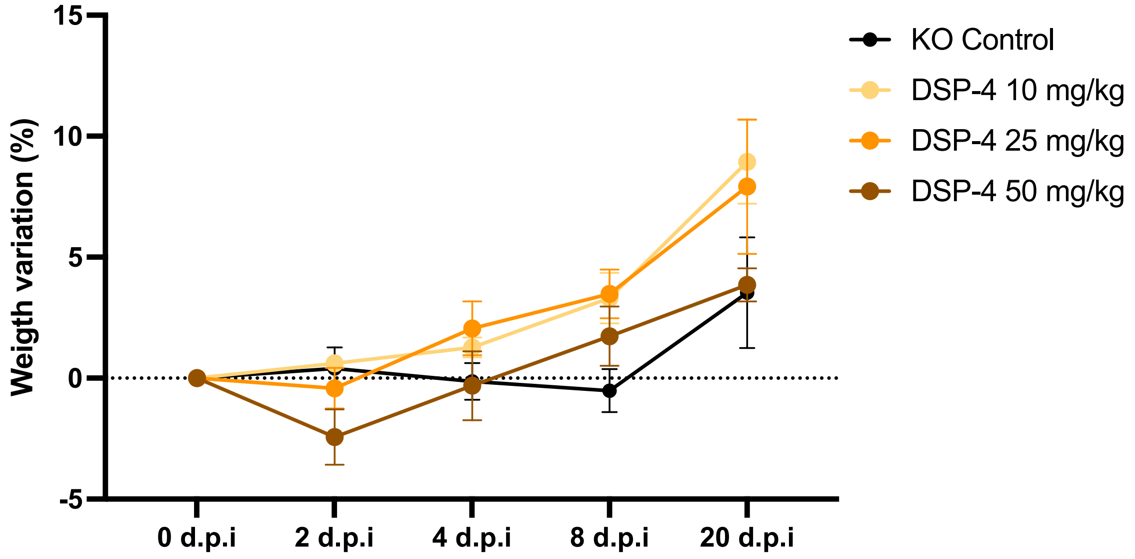

Figure S1. Body weight variation following N-(2-chloroethyl)-N-ethyl-2-bromobenzylamine (DSP-4) (C8417, Sigma-Aldrich, St. Louis, MO) lesion. DSP-4 was systemically injected by intraperitoneal injection (i.p.) at defined dosages (10-, 25- and 50mg/kg). Abbreviations: KO (knockout), d.p.i. (Days Post Injection).
